# Peroxisome Proliferator-Activated Receptors and Progression of Colorectal Cancer

**DOI:** 10.1155/2008/931074

**Published:** 2008-06-05

**Authors:** Dingzhi Wang, Raymond N. DuBois

**Affiliations:** ^1^Department of Medicine, Vanderbilt University Medical Center, Nashville, TN 37232, USA; ^2^Departments of Gastrointestinal Oncology and Cancer Biology, MD Anderson Cancer Center, The University of Texas, Houston, TX 77030-4009, USA

## Abstract

The peroxisome proliferator-activated receptors (PPARs) are members of the nuclear hormone receptor superfamily. These receptors are also ligand-dependent transcription factors responsible for the regulation of cellular events that range from glucose and lipid homeostases to cell differentiation and apoptosis. The importance of these receptors in lipid homeostasis and energy balance is well established. In addition to these metabolic and anti-inflammatory properties, emerging evidence indicates that PPARs can function as either tumor suppressors or accelerators, suggesting that these receptors are potential candidates as drug targets for cancer prevention and treatment. However, conflicting results have emerged regarding the role of PPARs on colon carcinogenesis. Therefore, further investigation is warranted prior to considering modulation of PPARs as an efficacious therapy for colorectal cancer chemoprevention and treatment.

## 1. INTRODUCTION

Understanding the biology of intestinal epithelial cells may reveal the molecular pathogenesis of a number of digestive diseases. One such disease, colorectal cancer (CRC), leads to significant cancer-related morbidity and mortality in most industrialized countries. Initiation and progression of CRC are a complex process that results from the loss of the normal regulatory pathways that govern a balance between epithelial cell proliferation and death. For example, alterations in multiple pathways such as Wnt/APC, COX-2, and Ras are known to play major roles in CRC progression. The standard treatment for advanced malignancies has improved greatly over the past decade but is still not satisfactory. Therefore, significant effort has been exerted to identify novel drug targets for both the prevention and treatment of this disease. One group of compounds found to decrease the risk of colorectal cancer includes nonsteroidal anti-inflammatory drugs (NSAIDs), which target the cyclooxygenase enzymes (COX-1 and COX-2). However, prolonged use of high doses of these inhibitors (except for aspirin) is associated with unacceptable cardiovascular side effects [1–3]. Thus, it is now crucial to develop more effective chemopreventive agents with minimal toxicity and maximum benefit.

Dietary fat intake is an environmental factor that is associated with some human diseases such as diabetes, obesity, and dyslipidemias. Some nuclear hormone receptors play a central role in regulating nutrient metabolism and energy homeostasis. These nuclear receptors are activated by natural ligands, including fatty acids and cholesterol metabolites. Among these receptors, special attention has been focused on the members of the peroxisome proliferator-activated receptors (PPARs) family, which were initially identified as mediators of the peroxisome proliferators in the early 1990s [4]. PPARs play a central role in regulating the storage and catabolism of dietary fats via complex metabolic pathways, including fatty acid oxidation and lipogenesis [5]. To date, three mammalian PPARs have been identified and are referred to as PPAR*α* (NR1C1), PPAR*δ*/*β* (NR1C2), and PPAR*γ* (NR1C3). Each PPAR isotype displays a tissue-selective expression pattern. PPAR*α* and PPAR*γ* are predominantly present in the liver and adipose tissue, respectively, while PPAR*δ* expresses in diverse tissues [6]. In common with other members of the type II steroid hormone receptor superfamily, PPARs are ligand-dependent transcription factors and form heterodimers with another obligate nuclear receptors, such as retinoid X receptors (RXRs) [4, 7, 8]. Each PPAR-RXR heterodimer binds to the peroxisome proliferator responsive element (PPRE) located in the promoter region of responsive genes.

It is well established that modulation of PPAR activity maintains cellular and whole-body glucose and lipid homeostases. Hence, great efforts have been made to develop drugs targeting these receptors. For example, PPAR*γ* synthetic agonists, rosiglitazone and pioglitazone, are antidiabetic agents which suppress insulin resistance in adipose tissue. The antiatherosclerotic and hypolipidemic agents including fenofibrate and gemfibrozil are PPAR*α* synthetic agonists that induce hepatic lipid uptake and catabolism. Genetic and pharmacological studies have also revealed important roles of PPAR*δ* in regulating lipid metabolism and energy homeostasis. Genetic studies indicate that overexpression of constitutively active PPAR*δ* in mouse adipose tissue reduced hyperlipidemia, steatosis, and obesity induced by either genetics or a high-fat diet. In contrast, PPAR*δ* null mice treated in similar fashion exhibited an obese phenotype [9]. Pharmacologic studies demonstrate that the PPAR*δ* selective-agonist (GW501516) attenuated weight gain and insulin resistance in mice fed with high-fat diets [10] and increased HDL-C while lowering tryglyceride levels and insulin in obese rhesus monkeys [11]. Furthermore, preclinical studies revealed that PPAR*δ* agonists diminished metabolic derangements and obesity through increasing lipid combustion in skeletal muscle [12]. These results suggest that PPAR*δ* agonists are potential drugs for use in the treatment of dyslipidemias, obesity, and insulin resistance. Therefore, the PPAR*δ* agonist (GW501516) is currently in phase III clinical trials to evaluate its use for treatment of patients with hyperlipidemias and obesity. However, recent studies showing that some agonists of PPARs promote carcinogenesis in animal models have raised concerns about using these agonists for the treatment of metabolic diseases. For example, long-term administration of a PPAR*α* agonist induces the development of hepatocarcinomas in mice but not in PPAR*α* null animals, conclusively demonstrating that PPAR*α* mediates these effects in promoting liver cancer [13]. Furthermore, the PPAR*δ* agonist (GW501516) accelerates intestinal polyp growth in Apc^Min/+^ mice [14, 15]. These results raise concerns for developing this class of agents for human use and support the rationale for developing PPAR*δ* antagonists as chemopreventive agents.

## 2. PPARs AND COLORECTAL CANCER

Significant effort has been concentrated on deducing the role of PPARs in CRC and other cancers. A large body of evidence indicates that PPAR*γ* serves as a tumor suppressor. Contradictory evidences suggest that PPAR*δ* can act as either a tumor suppressor or tumor promoter. A few evidences support a role of PPAR*α* in CRC.

### 2.1. PPAR*α*

Although the tumor-promoting effects of PPAR*α* in hepatocarcinomas are clear, less is known about the role of PPAR*α* in human tumors. Generally, activation of PPAR*α* by exogenous agonists causes inhibition of tumor cell growth in cell lines derived from CRC, melanoma, and glial brain tumors [16–18]. There is no evidence showing that PPAR*α* expression is elevated in human cancers.

### 2.2. PPAR*γ*

The prominent role of PPAR*γ* in regulating cellular differentiation prompted a great effort to investigate the function of PPAR*γ* in cancer field. While PPAR*γ* is elevated in CRC [19], suggesting that this receptor may contribute to tumor biology, studies of PPAR*γ* mutation in CRC from humans, animals, and cultured cells produced controversial results. One study showed that 8% of primary human colorectal tumors had a loss of function mutation in one allele of the PPAR*γ* gene [20]. Recent data revealed that a Pro12Ala (P12A) polymorphism in the PPAR*γ* gene is associated with increased risk of CRC [21, 22]. These results suggest a putative role for this receptor as a tumor suppressor. In contrast, another study showed that mutant PPAR*γ* gene has not been detected in human colon tumor samples and CRC cell lines, suggesting that PPAR*γ* mutations in human CRC is a rare event [23].

In vitro studies show that activation of PPAR*γ* results in growth arrest of colon carcinoma cells through induction of cell-cycle arrest or/and apoptosis. Several potential downstream targets of PPAR*γ* for mediating antitumor effects of PPAR*γ* have been identified in various cancer cell types. Activation of PPAR*γ* negatively regulates cell cycle progression by modulating a number of cell cycle regulators: (1) inhibiting E2F activity in transformed adipogenic cells [24], (2) Rb hyperphosphorylation in vascular smooth muscle cells and pituitary adenoma cells [25, 26], (3) cyclin D1 expression in Ras-transformed intestinal epithelial cells, pancreatic, or breast cancer cells [27–29], and (4) inducing CDK inhibitor expression such as p18, p21, and p27 in hepatoma cells [30]. Activation of PPAR*γ* has also been reported to inhibit tumor cell growth by upregulation of the transcriptional repressor TSC22 in colon cancer cells [31] and GADD153 in nonsmall-cell lung carcinoma cells [32]. PPAR*γ* agonists induce apoptosis by induction of PTEN expression in pancreatic, breast, and colon cancer cells [33] and inhibition of NF*κ*B and Bcl-2 expression in colon cancer cells [34]. Moreover, PPAR*γ* exhibits antiangiogenic effects by inhibiting VEGF expression in tumor cells and VEGF receptors in endothelial cells [35, 36]. It has also been reported that PPAR*γ* agonists suppress tumor cell invasion in colon and breast cancer cells by downregulation of matrix metalloproteinase-7 (MMP-7) and induction of MMP inhibitors [37, 38]. In addition, the ability of PPAR*γ* to suppress tumor growth is also through inhibiting APC/*β*-catenin and COX-2/PGE_2_ signaling pathways, which are pivotally involved in colon carcinogenesis [39–42].

However, the role of PPAR*γ* in colorectal cancer progression is controversial because there are conflicting results in mouse models of colon cancer. Although PPAR*γ* agonists inhibit colorectal carcinogenesis in xenograft models and in the azoxymethane (AOM)-induced colon cancer model [43, 44], these drugs are reported to have both tumor-promoting and tumor-inhibiting effects in a mouse model for familial adenomatous polyposis, the Apc^Min/+^ mouse. It has been reported that administration of PPAR*γ* agonists significantly increases the number of colon adenomas in the Apc^Min/+^ mice [45–47] and even in wild-type C57BL/6 mice [48]. However, other studies show that treatment of 2 different Apc-mutant models (Apc^Min/+^ and Apc^Δ1309^) with the PPAR*γ* agonist pioglitazone resulted in reduction in the number of both small and large intestinal polyps in a dose-dependent manner [49, 50]. These paradoxical observations appear to have been resolved by genetic studies showing that the heterozygous disruption of PPAR*γ* is sufficient to increase tumor number in AOM-treated mice and that intestinal-specific PPAR*γ* knockout promotes tumor growth in Apc^Min/+^ mice [39, 51]. These genetic evidences support the hypothesis that PPAR*γ* serves as tumor suppressor in colorectal cancer. One possible explanation for the differences in phenotype caused by pharmaceutical versus genetic manipulation of PPAR*γ* in mouse models may be due to the PPAR*γ*-independent effect of the agonist drugs, drug doses used, and animal models employed. This controversial extends beyond CRC. For example, data are conflicting from different animal models of breast cancer as well. PPAR*γ* agonist suppresses NMU-induced mammary carcinomas [52]. However, overexpression of a constitutively active form of PPAR*γ* accelerates mammary gland tumor development in MMTV-PyV transgenic mice [53].

### 2.3. PPAR*δ*

PPAR*δ* has been shown to play an important role in embryo implantation [54], atherogenic inflammation [55], regulating cell survival in the kidney following hypertonic stress [56], and skin following wound injury [57, 58]. The role of PPAR*δ* in colorectal carcinogenesis is more controversial than that of PPAR*γ*. The first evidence linking the PPAR*δ* to carcinogenesis actually emerged from studies on gastrointestinal cancer. PPAR*δ* is elevated in most human colorectal cancers and in tumors arising in the Apc^Min/+^ mice, and AOM-treated rats [59, 60]. Importantly, the PPAR*δ* proteins are accumulated only in human CRC cells with highly malignant morphology [61]. Downregulation of PPAR*δ* is correlated with antitumor effects of dietary fish oil/pectin in rats treated with radiation and AOM [62]. PPAR*δ* was identified as a direct transcriptional target of APC/*β*-catenin/Tcf pathway and as a repression target of NSAIDs [59, 63]. A case-control study in a large population showed that the protective effect of NSAIDs against colorectal adenomas was reported to be modulated by a polymorphism in the PPAR*δ* gene [64]. PPAR*δ* expression and activity are also induced by oncogenic K-ras [65]. In addition, COX-2-derived PGl_2_ directly transactivates PPAR*δ* [60], and COX-2-derived PGE_2_ indirectly induces PPAR*δ* activation in CRC, hepatocellular carcinoma, and cholangiocarcinoma cells [66–68]. These studies indicate that PPAR*δ* is a focal point of cross-talk between these signaling pathways.

In a murine xenograft cancer model, the disruption of both PPAR*δ* alleles in human HCT-116 colon carcinoma cells decreased tumorigenicity, suggesting that activation of PPAR*δ* promotes tumor growth [69]. However, PPAR*δ* has been reported to have both tumor-promoting and tumor-inhibiting effects based on conflicting data obtained from mouse models of colon cancer. For example, activation of PPAR*δ* by a selective synthetic PPAR*δ* agonist (GW501516) or a PPAR*δ* endogenous activator (PGE_2_) accelerates intestinal adenoma growth in Apc^Min/+^ mice by promoting tumor cell survival [14, 66]. A subsequent genetic study showed that deletion of PPAR*δ* attenuates both small and large intestinal adenoma growth, and PPAR*δ* is required for the tumor-promoting effects of PPAR*δ* ligand (GW501516) and PGE_2_ in Apc^Min/+^ mice [15, 66]. Another study showed that loss of PPAR*δ* in Apc^Min/+^ mice significantly reduced growth of tumors larger than a diameter of 2 mm, even though PPAR*δ* deficiency did not affect overall tumor incidence [70]. In contrast to these reports suggesting that PPAR*δ* serves as tumor accelerator, recent conflicting reports show that PPAR*δ* deficiency enhances polyp growth in Apc^Min/+^ and AOM-treated mice in the absence of exogenous PPAR*δ* stimulation [71, 72]. Moreover, a PPAR*δ* ligand (GW0742) inhibits colon carcinogenesis in AOM-treated mice but promotes small intestinal polyp growth in Apc^Min/+^ mice [73].

One explanation for these disparate results may be due to differences in the genetic background of Apc^Min/+^ mice, animal breeding, or possibly to differences in the specific targeting strategy employed to delete PPAR*δ*. For example, the average number of polyps in 13-week old Apc^Min/+^ mice on a C57BL/6 genetic background is about 50, while the polyp number in Apc^Min/+^ mice on a mixed-genetic-background (C57BL/6 × 129/SV) is about 120. Our results also show that the breeding strategy affects the number and size of polyps in mice even on the same genetic background. Mice generated by breeding female PPAR*δ* ^−/−^/Apc^Min/+^ with male PPAR*δ* ^−/−^/Apc^+/+^ exhibit increased adenoma number with a larger average size than those obtained by breeding female PPAR*δ* ^−/−^/Apc^+/+^ with male PPAR*δ* ^−/−^/Apc^Min/+^. Finally, the PPAR*δ* null mice we studied were obtained from Beatrice Desvergne in Switzerland. These mice were generated by deleting exons 4 and 5 encoding the DNA binding domain [74], while Peters group generated the PPAR*δ* knockout mice by inserting a neomycin resistance cassette into the last exon (exon 8) [75]. It has been suggested that the strategy employed to disrupt PPAR*δ* by the Peters group might have led to a hypomorphic allele, which retains some aporeceptor function, thus making it difficult to correctly interpret their results. Indeed, conflicting results in the context of embryonic lethality have also been observed from these two PPAR*δ* mutant mouse strains [74, 75]. To further clarify the role of PPAR*δ* in colorectal tumorigenesis, it is important to investigate the role of PPAR*δ* in animal models that are dependent on activation of other oncogenes or disruption of other tumor suppressors to verify our conclusions that activation of PPAR*δ* is proneoplastic.

Studies in other types of cancer also support the hypothesis that PPAR*δ* serves as a tumor accelerator. A selective PPAR*δ* agonist (GW501516) has been shown to stimulate proliferation of human breast, prostate, and hepatocellular carcinoma cells [68, 76, 77]. In a xenograft model, blocking PPAR*δ* activation reduced ovarian tumor growth [78]. PPAR*δ* knockout mice exhibited significant impaired angiogenesis and tumor growth after these mice were injected s.c. with mouse Lewis lung carcinoma and melanoma cells [79]. In a mouse mammary tumor model, treatment with the PPAR*δ* agonist (GW501516) accelerated tumor formation, while a PPAR*γ* agonist (GW7845) delayed tumor growth [80]. Taken together, the role of PPAR*δ* in cancer biology remains unclear.

## 3. SUMMARY

**Figure 1:**
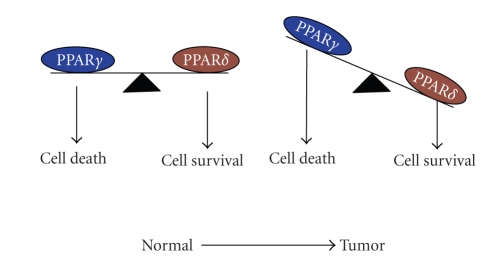
A potential model for PPARs regulating colorectal tumor growth.

Despite extensive research on both PPAR*γ* and PPAR*δ* in CRC, the role of these receptors remains highly controversial in this disease. Emerging evidence demonstrates that cooperative interactions between Wnt, COX-2, and PPARs signaling pathways can initiate cellular transformation and promote progression of colorectal cancer. These studies provide support for evaluating the efficacy of PPAR*δ* antagonists for cancer prevention and/or treatment. We propose a potential working model as a useful starting point for future studies (see Figure 1).
